# The effects of mobile phone addiction on bedtime procrastination in university students: the masking effect of physical activity and anxiety

**DOI:** 10.1186/s40359-024-01899-z

**Published:** 2024-07-17

**Authors:** Shuqiao Meng, Yu Zhang, Lingling Tang, Meng Zhang, Wenjing Tang, Nzubechi Onyebuchi, Yahui Han, Shanshan Han, Bo Li, Wenxia Tong, Xiaoyu Ge

**Affiliations:** 1 Department of Physical Education, Xidian University, Xi ’an, 710126 Shaanxi Province China; 2 School of Physical Education, Henan University, Kaifeng, 475001 Henan Province China; 3 College of Physical Education, Yangzhou University, Yangzhou, 225009 Jiang Su Province China; 4 Department of Physical Education, Henan University, Kaifeng, 475001 Henan Province China; 5 Institute of Sports Science, Kyunggi University, Suwon, 449701 South Korea; 6 Institute of Sports Science, Nantong University, Nantong, 226019 Jiangsu Province China; 7 Sports Department of Northwestern Polytechnical University, Xi’an, 710072 Shaanxi Province China

**Keywords:** Mobile phone addiction, Bedtime procrastination, Physical activity, Anxiety, Chain mediation

## Abstract

**Purpose:**

Good sleep is one of the necessary conditions to ensure the normal performance of the physiological and psychological functions of college students. This study aimed to explore the relationship between mobile phone addiction and bedtime procrastination among Chinese college students and the mediating mechanisms of physical exercise and anxiety between the two, with a view to seek ways to prevent and intervene in college students’ sleep procrastination and improve their sleep quality.

**Methods:**

Using SPSS 29.0 analysis with Bootstrap’s method, 3,800 first-year students, sophomores, and juniors were given the Mobile Phone Addiction Tendency Scale, Bedtime Procrastination Scale, Physical Activity Scale, and Anxiety Scale. The results of the analyses included mediation tests and effect analyses of anxiety and physical activity.

**Results:**

The correlation analysis revealed significant positive correlations between mobile phone addiction and bedtime procrastination (*r* = 0.149, *p* < 0.01) as well as anxiety (*r* = 0.497, *p* < 0.01). Additionally, there was a significant negative correlation between mobile phone addiction and physical activity (*r* = -0.447, *p* < 0.01). Physical activity was also found to have significant negative correlations with anxiety (*r* = -0.506, *p* < 0.01) and bedtime procrastination (*r* = -0.424, *p* < 0.01). Furthermore, anxiety showed a significant positive correlation with bedtime procrastination (*r* = 0.334, *p* < 0.01). Physical activity and anxiety acted as substantial mediators between mobile phone addiction and nighttime procrastination. Both mediators had considerable masking effects, with the mediating effect amounting to 50.3% and 25.1%, respectively. Physical exercise and anxiety played a chain mediating role between mobile phone addiction and bedtime procrastination, and the masking effect was also significant, with a mediating effect size of 13.4%.

**Conclusions:**

This study reveals the special characteristics of the influencing factors and pathways of bedtime procrastination in this group of college students, providing targeted evidence for the prevention and intervention of bedtime procrastination in college students. It also has an important reference value for the effects of exercise and comprehensive intervention to improve bedtime procrastination and enhance the quality of sleep in college students.

## Introduction

In reference to the 52nd " Statistical Report on Internet Development in China " released by China Internet Network Information Centre (CNNIC), it shows that as of June 2023, the number of mobile phone netizens in China has reached 1.076 billion, accounting for 99.7% of the overall size of netizens (1.079 billion), and (21.7%) [1] students accounted for the extensive proportion of netizen groups .

Teen’s daily lives and studies have been incorporated with mobile phones, which are the carriers of mobile Internet development. These devices are gradually altering the way teenagers live and behave. Mobile phone addiction refers to the excessive use of and dependence on mobile phones that leads to problematic psychology or behavior in daily life, known as mobile phone dependence, mobile phone problematic use, and mobile phone addiction [2]. Previous studies have shown that mobile phone addiction can negatively affect college students’ academic performance, mental health, physical health, and social functioning, such as reducing academic engagement [3, 4]. inducing depression [5–7],and procrastination [8, 9]. An in-depth exploration of the influence mechanism and psychological mechanism of mobile phone addiction in college students is conducive to guiding college students to use their functional mobile phones reasonably, which is of great significance to the prevention and mobile phone addiction.

Sleep procrastination refers to the behavior of an individual who habitually delays his or her scheduled bedtime in the absence of an external obstacle [10]. Bedtime procrastination Research has found that sleep procrastination can lead to sleep deprivation, difficulty in falling asleep, increased negative emotions, and in the long run can lead to physical and mental disorders such as disturbed sleep patterns, anxiety, and decreased immune function [11, 12]. The university stage is an essential transition period in life development, adequate or quality sleep is the foundation guarantee for life normality and study, but poor sleep behavior and habits will have many adverse effects on their sleep, body, and mind [13, 14]. According to the immersion theory, an individual can feel immersed when using Internet media such as mobile phones and computers, therefore involuntarily immersing themselves in the pleasurable experience brought by the Internet, resulting in the loss of self-consciousness and the change of the sense of time, and delaying the time when they should have gone to bed [15]. Based on this theory, this present study hypothesized Mobile phone addiction positively predicts sleep-delaying behavior in college students(H1).

Physical exercise involves physical movement as the content and means to promote an individual’s physical and mental health development. It can vary in intensity, frequency and lenght [16]. According to the Theories of Usage and Gratification, when individuals use the Internet, their psychological needs, such as social interaction and entertainment, will be satisfied to varying degrees, and this sense of satisfaction will motivate individuals to use mobile phones and other network devices more frequently, which in the long run will lead to the problem of mobile phone addiction [17]. Multiple studies have shown that independent and individualistic college students are more likely to develop mobile phone addictions [18]. Research has pointed out that appropriate physical activity can enhance the dopaminergic signaling ability of the human body, reduce the negative emotions of college students, satisfy their psychological needs, reduce the frequency of mobile phone use, and improve the phenomenon of mobile phone addiction [19–21]. Research on physical activity and sleep quality is more mature; regular physical activity can not only significantly improve sleep quality but also serve as a self-help therapy for individuals with sleep disorders, prompting them to enter sleep quickly and reducing the occurrence of sleep procrastination behavior [22–24]. It accordingly proposes that Physical exercise mediates the effect of mobile phone addiction on college students’ sleep procrastination behavior (H2).

Studies have shown that mobile phone addiction significantly predicts anxiety, and anxiety significantly predicts mobile phone addiction, with the two influencing and causally affecting each other [2] The two are mutually reinforcing. Firstly, the internet access provided by mobile phones makes anxious individuals more likely to become addicted to mobile phones.Davis’ (2001) Cognitive-Behavioral Model of Pathological Internet Use suggests that distal factors contribute to internet addiction through proximal non-adaptive cognitions [25]. Anxiety is a distal factor that affects Internet addiction. Secondly, mobile phones attract people not only because of their functionality but also because of their ability to provide deep psychological comfort to individuals. Mobile phones are like pacifiers for babies; their portability, privacy, and good sense of tactile experience can effectively relieve anxiety [26]. Studies have shown that the higher the level of anxiety, the more severe the sleep problems, which can be manifested in more difficulty falling asleep and waking up multiple times during the night [27]. The theoretical model of the relationship between anxiety and sleep suggests that sources of anxiety can directly affect sleep problems and that cognitive responses, depressed mood, etc. are important mediating variables that explain the relationship between sources of anxiety and sleep [11, 14, 28]. Sleep procrastination behavior is one of the key elements determining the quality of each person’s sleep, and anxiety is a significant mediating factor between mobile phone addiction and other variables [29]. Based on this, the study proposed that anxiety mediates the relationship between the procrastination of sleep by college students and the effect of mobile phone addiction(H3).

Anxiety and physical activity may play both separate and chained-mediating effects in mobile phone addiction and bedtime procrastination among college students. It has been shown that physical activity significantly and negatively predicts anxiety [30]. Based on this, the present study hypothesized Anxiety and physical activity play a chain mediating role in the relationship between mobile phone addiction and sleep procrastination among college students(H4).

Through an assessment of the factors that contribute to college students’ sleep issues from the stand point of mobile phone addiction and analysis of the means and condition of influence, we can provide empirical evidence and useful guidance for safeguarding and improving college students’ sleep quality, which can have more evident preventive and intervention outcomes. However, most of the current research focuses on the direct relationship between mobile phone addiction and sleep quality but lacks the relationship between mobile phone addiction and sleep procrastination, as well as the mechanism of influence between the two. Therefore, the present study will explore the effects of mobile phone addiction on bedtime procrastination and its mechanism of action among college students.

## Research methodology

### Subjects

The formal questionnaire was distributed using a convenience sampling method by generating a link (https://www.wjx.cn) through the Questionnaire Star platform, and then the survey was conducted at Xi’an University of Electronic Science and Technology (XUET), Xi’an University of Technology (XUT), Xi’an Jiaotong University (XJTU), Shaanxi Normal University (SNU), including other colleges and universities through China’s social media platforms, e.g., WeChat and QQ. The survey lasted 10 weeks, from October 2, 2023, to December 4, 2023. After removing the incorrect questionnaires, 3800 recovered questionnaires were eventually reduced to 3599 valid questionnaires. (e.g., answering questions too uniformly or taking too little time to fill them out, etc.), with a data recovery rate of 94.7%. Among them, in 1958 (55.2%) were male and 1614 (44.8%) were female; 1261 (35.0%) were freshmen, 2212 (61.5%) were sophomores, and 126 (3.5%) were juniors. Age was 19.12 ± 1.05 years.

### Research tools

#### Physical activity scale

A revised version of the Physical Activity Rating Scale (PARS-3) developed by T.C. Leung was selected [31]. Three factors were used to rate participants’s levels of physical activity: frequency, duration, and intensity of exercise. Calculation formula: physical activity = exercise frequency score × (exercise duration score − 1) × exercise intensity score. The scale was scored on a 5-point Likert scale, with 5 options for each question scored on a scale of 1 to 5. The total score ranged from 0 to 100, with higher scores indicating higher levels of physical activity participation. The low-level physical activity group was ≤ 19 points, the moderate-level physical activity group was between 20 and 42 points, and the high-level physical activity group was ≥ 43 points. The Cronbach’s alpha value for this scale in this study was 0.821.

#### Mobile phone addiction scale

The mobile phone addiction tendency scale for college students developed by Xiong Jie et al. (2012) was used [32] to assess the degree of individual mobile phone addiction. The scale consists of 16 questions, including withdrawal symptoms, prominent behavior, social comfort, and mood change.With a minimum score of 16 and a maximum score of 80, the scale is rated on “Z” 5-point Likert scale, where 1 indicates “very non-compliant” and 5-means “very compliant.”The higher the score, the more likely a person is to be addicted to a mobile phone. The scale used in this study has a Cronbach’s alpha of 0.923.

#### Anxiety scale

A simplified Chinese version of the Depression-Anxiety-Stress Scale (DASS-21), based on the Depression-Anxiety-Stress Scale (DASS), was used by the Chinese scholars Gong Xu and Xie Xiyao Lovibond (1995) and others [33]. The Depression Anxiety Stress Scale (DASS-21) is a simplified Chinese version of the Depression Anxiety Stress Scale (DASS) [34]. The DASS-21 is a 21-item scale with three dimensions: depression, anxiety, and stress.The anxiety scale, which consists of seven questions overall, was employed in this investigation. A 4-point Likert scale ranging from 0 to 3 was used to describe the extent of an individual’s recent (“within the past week”) negative emotional experiences or physiological reactions, with 0 representing a complete lack of conformity, and 3 representing strong conformity, with higher scores indicating higher levels of anxiety in the individual. The Cronbach’s alpha for anxiety in this study was 0.841.

#### Chinese version of the Bedtime Procrastination Scale

The Chinese version of the Bedtime Procrastination Scale (BPS) revised by Ma Xiaohan et al. was used [35] There are nine items on the scale. The scale is rated on a 5-point scale from “1 = almost never” to “5 = almost always”, with higher total scores indicating more severe sleep procrastination. In this study, the Cronbach’s alpha of the scale was 0.982.

### Data processing

SPSS 29.0 software was used for entry and descriptive statistics. K-S nonparametric tests, reliability analyses, and exploratory factor analyses were used to test the normal distribution of data and instrument reliability and validity. For standardized data, the Mann-Whitney U-test was used to examine gender differences on a variable-by-variable basis, and the Kruskal-Wallis H-test was used to examine the grade differences on a variable-by-variable basis. Count data were expressed as component ratios (%), measures as x-±s, and correlations between variables were analyzed using Pearson correlation analysis. Hayes (2013) was used to [36] SPSS macro program PROCESS 4.1 plug-in developed for chained mediated effects model analysis and testing. Parameter estimate was performed using the the Bootstrap method of bias reduction, and 95% confidence intervals were provided. At *p* < 0.05, differences were deemed statistically significant.

## Results and analyses

### Common methodology bias control and testing

Based on the fact that the research data was collected using a questionnaire, the results may be affected by common methodological bias [37] The test was conducted using the Harman one-way test, which shows that there are 10 factors with a root cause of greater than 1, of which the first factor explains the cumulative variance of the factors.

Harman’s one-way test, revealing that ten factors had eigenroots greater than 1 was used. The cumulative variance explained by the first factor was 22. 62%, which is less than the critical value of 40%. It indicates that there is no serious common method bias in this study.

### Group difference analysis of mobile phone addiction, bedtime procrastination, physical activity and anxiety

The Mann-Whitney U-test for gender and the Kruskal-Wallis H-test for grade showed (see Tables 1 and 2) that the gender difference, and grade difference for mobile phone addiction, bedtime procrastination, physical activity and anxiety were significant (*p* < 0.05). Mobile phone addiction (37.25 ± 16.47), bedtime procrastination (3.27 ± 1.69), and anxiety (5.30 ± 5.41) were higher in male university students than in female students, while physical activity (17.36 ± 19.21) was lower in male university students than in female university students (17.36 ± 19.21). In terms of grade, mobile phone addiction (39.32 ± 13.91, 34.06 ± 17.05, 31.03 ± 15.03), bedtime procrastination (3.29 ± 1.26, 3.28 ± 1.80, 3.17 ± 2.14) and anxiety (4.98 ± 4.40, 4.87 ± 5.40, 4.00 ± 6.02) in decreasing order, while physical activity along with showed an increasing trend (17.47 ± 18.79, 17.96 ± 20.15, 20.88 ± 20.93).

**Table 1:** Mann-Whitney U test for gender and Kruskal-Wallis H test for grade (*M ± SD*)

	Mobile phone addiction	Bedtime procrastination	Physical exercise	Anxiety
Mann-Whitney U	1,320,595	1380599.5	1430591.5	1,327,696
Wilcoxon W	2,580,673	2640677.5	3225156.5	2,587,774
*Z*	-6.176	-4.199	-2.454	-5.973
Asymptotic significance (two-tailed)	< 0.001	< 0.001	0.014	< 0.001
chi-square (math.)	22.020	34.160	8.312	32.885
*df*	2	2	2	2
Progressive significance	< 0.001	< 0.001	0.016	< 0.001
Male	37.25 ± 16.47	3.27 ± 1.69	17.36 ± 19.21	5.30 ± 5.41
Female	32.94 ± 16.05	3.22 ± 1.71	18.65 ± 20.56	4.36 ± 4.90
first-year student	39.32 ± 13.91	3.29 ± 1.26	17.47 ± 18.79	4.98 ± 4.40
Sophomore	34.06 ± 17.05	3.28 ± 1.80	17.96 ± 20.15	4.87 ± 5.40
Junior	31.03 ± 15.03	3.17 ± 2.14	20.88 ± 20.93	4.00 ± 6.02

### Descriptive statistics and correlation analysis

The results of the descriptive statistics and correlation analysis of the variables are shown in Table 2. As shown in Table 2, mobile phone addiction was significantly positively correlated with bedtime procrastination and anxiety (*r* = 0.149, *p* < 0.01; *r* = 0.497, *p* < 0.01) and significantly negatively correlated with physical activity ( *r* =-0.447, *p* < 0.01 ); physical activity was significantly negatively correlated with anxiety and bedtime procrastination were significantly negatively correlated (*r* =-0.506, *r* < 0.01; *r* =-0.424, *p* < 0.01); and anxiety was significantly positively correlated with bedtime procrastination (*r* = 0.334, *p* < 0.01). Gender was significantly negatively correlated with mobile phone addiction, bedtime procrastination, and anxiety (*r* =-132, *r* < 0.01; *r* =-0.012, *p* < 0.01; *r* =-0.090, *p* < 0.01) and significantly positively correlated with physical activity (*r* = 0.032, *p* < 0.01). The results of the correlation analysis were generally consistent with theoretical expectations, providing a feasible basis for subsequent hypothesis testing.

**Table 2:** Descriptive statistics and correlation analysis results

	M	SD		1	2	3	4	5	6	7	8
1.Gender	1.46	0.499		1							
2.Mobile phone addiction	35.25	16.42		− 0.131^**^	1						
3.Withdrawal symptoms	15.51	7.2		− 0.121^**^	0.933^**^	1					
4.Prominent behavior	9.27	4.73		− 0.121^**^	0.953^**^	0.950^**^	1				
5.Social comfort	7.71	3.43		− 0.114^**^	0.947^**^	0.962^**^	0.959^**^	1			
6.Mood change	6.95	2.97		− 0.119^**^	0.981^**^	0.935^**^	0.977^**^	0.963^**^	1		
7.Bedtime procrastination	3.25	1.69		− 0.012^**^	0.149^**^	0.166^**^	0.251^**^	0.192^**^	0.223^**^		
8.Anxiety	4.86	5.2		− 0.090^**^	0.479^**^	0.510^**^	0.598^**^	0.515^**^	0.543^**^	0.334^**^	1
9.Physical exercise	17.95	19.86		0.032^**^	− 0.447^**^	− 0.419^**^	− 0.509^**^	− 0.485^**^	− 0.533^**^	− 0.424^**^	− 0.506^**^

Note: *n* = 3599; all variables in the model were standardised;^**^p < 0.01; gender: 1 = male, 2 = female

### Analysis of chain-mediated effects of physical activity and anxiety

With mobile phone addiction as the independent variable, bedtime procrastination as the dependent variable, and physical exercise and anxiety as the mediating variables, regression analysis of the chain mediating effect of physical exercise and anxiety between mobile phone addiction and bedtime procrastination was conducted using SPSS29.0 and PROCESS4.1 plug-in controlling for gender, and the results are shown in Table 3. The following are the precise steps: in the first step, bedtime procrastination was used as the dependent variable, and gender was entered as the control variable to control the possible effect of gender on bedtime procrastination. In the second step, mobile phone addiction was input to establish a regression model to examine the total effect of mobile phone addiction on bedtime procrastination after controlling the variable, and it was found that mobile phone addiction could significantly and positively predict bedtime procrastination (β = 0.006, *p* < 0.001), indicating that research hypothesis H1 was established. In the third step, physical activity and anxiety variables were sequentially added to the model to test whether the two were mediating variables for mobile phone addiction and bedtime procrastination and whether there was a chain mediating effect. The results found that Internet addiction significantly negatively predicted bedtime procrastination of college students (β = -0.050, *p* < 0.001), mobile phone addiction could significantly negatively predict physical exercise (β = -0.581, *p* < 0.001), and physical exercise significantly negatively predicted bedtime procrastination of college students (*β* = -0.050, *p* < 0.001) and anxiety (*β* = 0.116, *p* < 0.001), mobile phone addiction significantly and positively predicted anxiety (*β* = 0.089, *p* < 0.001), and anxiety significantly and positively predicted college students’ bedtime procrastination (*β* = -0.104, *p* < 0.001). The above coefficients indicate that physical activity mediates between mobile phone addiction and bedtime procrastination, proving H2; anxiety mediates between mobile phone addiction and bedtime procrastination, proving research hypothesized H3; and physical activity and anxiety chain mediate between mobile phone addiction and bedtime procrastination, proving H4.

**Table 3:** Regression analysis of variable relationships in the chained mediation model

Regression equation (*n* = 3599)		Overall fit index		Significance of regression coefficients		95 per cent confidence interval	
outcome variable	predictor variable	R^2^	F	*β*	t	LLCI	ULCI
Bedtime procrastination	Mobile phone addiction	0.002	3.919	0.006	-2.79	− 0.010	− 0.002
	Gender			0.0208	0.309	-1111	0153
Physical exercise	Mobile phone addiction	0.201	450.126	-0.545	-29.992	− 0.581	− 0.510
	Gender			-1.057	-1.765	-2.230	0.117
Anxiety	Mobile phone addiction	0.338	610.348	0.098	20.273	0.089	0.1079
	Physical exercise			− 0.096	-24.107	− 0.1037	− 0.0081
	Gender			− 0.395	-2.762	− 0.6749	− 0.1145
Bedtime procrastination	Mobile phone addiction	0.347	477.144	− 0.050	-25.667	− 0.054	− 0.046
	Physical exercise			− 0.046	-28.259	− 0.049	− 0.043
	Anxiety			0.128	20.206	0.116	0.141
	Gender			0.009	0.177	− 0.097	0.117

The mediation effect test was conducted according to the mediation effect test procedure introduced by Wen Zhonglin et al. [38]. The mediation effect test process introduced for the construct model of this study was conducted to test the mediation effect. 5000 Bootstrap samples were taken from the original data through repeated random sampling, and if the Bootstrap 95% confidence interval for the mediating effect did not contain 0, the mediating effect was indicated to be significant. In the first step, the total effect was examined, the presence of which indicated the possibility of an indirect effect. The point estimate of the total effect for mobile phone addiction → bedtime procrastination is -0.0056 with a standard deviation of 0.002, and the Bootstrap 95% confidence interval for the total effect generated by this pathway is [-0.0096,-0.0015], which does not contain a 0. This indicates that the total mobile phone addiction → bedtime procrastination effect is significant. In the second step, the indirect effect was tested, and the presence indicated the existence of the mediating effect. The point estimate of the indirect effect of mobile phone addiction→physical exercise→sleep procrastination is 0.0252, the standard deviation is 0.002, and the Bootstrap 95% confidence interval of the total effect generated by this path is [-0.0539,-0.0463], which doesn’t contain 0, indicating that the mediating effect of this path is significant. The point estimate of the indirect effect of mobile phone addiction→anxiety→bedtime procrastination was 0.0126, with a standard deviation of 0.0026, and the Bootstrap95% confidence interval of the total effect produced by this path was [0.0084,0.0183], not including 0, indicating that the mediating effect of this path was significant; the point estimate of the indirect effect of mobile phone addiction→physical exercise→anxiety→bedtime procrastination was 0.0067, with a standard deviation of 0.002, and the Bootstrap95% confidence interval of the total effect generated by this path was [0.0055,0.0082], which did not contain 0, indicating that the chained mediation effect is significant. In the third step, check the direct effect, if it’s smaller than the total effect, but significant, there is a partial mediation effect; if it’s not significant, it’s a full mediation effect. The point estimate of the direct effect of mobile phone addiction → bedtime procrastination is -0.0501, the standard deviation is -0.002, and the Bootstrap 95% confidence interval of the total effect produced by this path is [-0.0539,-0.0463], which does not contain 0, indicating that the mobile phone addiction → bedtime procrastination direct effect is significant and smaller than the total effect, indicating that there is a partial mediation effect. In the fourth step, comparing the signs of direct and indirect effects, if the same sign indicates the existence of partial mediation effect, reporting the ratio of mediation effect to total effect; if not the same sign, it is a masking effect, reporting the absolute value of the ratio of indirect effect to direct effect reflecting the effect size of mediation effect. The mediating effects of the three pathways in this study have different signs (positive and negative) from the direct effect, indicating a masking effect for all three pathways [38]. The absolute value of the ratio of the indirect effect to the direct effect was used to reflect the effect size of the mediating effect according to the suggestion of Wen Zhonglin and Ye Baojuan. The absolute value of the ratio of the mediating effect to the direct effect for physical activity was 50.3%, the absolute value of the ratio of the mediating effect to the direct effect for anxiety was 25.1%, and the absolute value of the ratio of the chained mediating effect to the direct effect for physical activity and anxiety was 13.4%. Results are provided in Table 4.

**Table 4:** Tests of mediating effects of physical activity and anxiety

	Effect	Boot SE	95 per cent confidence interval		|Indirect effects/direct effects
			Boot LLCI	Boot ULCI	
Total effect	− 0.0056	0.0020	− 0.0096	− 0.0015	
Direct effect	− 0.0501	0.0020	− 0.0539	− 0.0463	
Total indirect effect	0.0445	0.0034	0.0387	0.0517	
Mobile phone addiction → Physical activity→Bedtime procrastination	0.0252	0.0013	0.0227	0.0278	50.3%
Mobile phone addiction → Anxiety → Bedtime procrastination	0.0126	0.0026	0.0084	0.0183	25.1%
Mobile phone addiction → Physical activity → Anxiety→Bedtime procrastination	0.0067	0.0007	0.0055	0.0082	13.4%
C1	0.0126	0.0030	0.0058	0.0082	
C2	0.0185	0.0014	0.0157	0.0211	
C3	0.0059	0.0021	0.0024	0.0211	

## Discussion

### Mobile phone addiction has a significant positive effect on bedtime procrastination of university students

The results of the study indicate that mobile phone addiction is correlated with college students’ bedtime procrastination, suggesting that individuals with a high level of mobile phone addiction have more serious bedtime procrastination, which is basically in line with the results of existing studies [39–41]. The basic characteristics of mobile phone addiction are excessive use of mobile devices and a lack of self-control. According to the displacement hypothesis of the Internet, an individual’s time is constant, the more time and energy an individual spends on using a mobile phone, the less time and energy is spent on other tasks (e.g., sleep, exercise, work, etc.), resulting in delayed completion of these activities and tasks. An overactive state of the brain before sleep is not conducive to falling asleep in a short period of time, and individuals playing their mobile phones for a long period of time before bedtime increases the activity level of the brain, which leads to sleep-delaying behaviors [42, 43]. In addition, the light from mobile phone screens inhibits the pineal gland from secreting melatonin, prolonging the time it takes to enter sleep [44, 45]. For mobile phone-addicted individuals, the time it takes to gain the health benefits of sleep is much longer than the time it takes to gain immediate psychological satisfaction and pleasure through mobile phone use. The former is far less attractive to the individual than the latter, which explains why mobile phone addicts tend to postpone sleep tasks [46] which leads to bedtime procrastination with respect to the individual, which also supports the Internet displacement hypothesis to some extent. Sleep procrastination may lead to sleep disruption and a reduction in subjective sleep duration, especially since high levels of sleep procrastination are exacerbated by the pressure to go to bed at the last minute and to get to sleep quickly. Neuroscience suggests that chronic poor sleep quality disrupts the natural cycle of different sleep stages and reduces metabolic activity in areas of the brain system (prefrontal cortex, limbic system, etc.), and that changes in this area affect psychological behaviors, such as depression, self-control, and decision-making, leading to depressed moods and increased responses to negative stimuli [47–50]. In summary, the results of this study suggest that mobile phone addiction and sleep procrastination may cause college students to experience more negative emotions and negative behaviors, both of which are important risk factors for their physical and mental health.

### Physical exercise mediates the relationship between mobile phone addiction and bedtime procrastination in university students

The study shows that physical activity plays a partial mediating role in mobile phone addiction and bedtime procrastination of college students (mediating effect size of 50.3%), indicating that mobile phone addiction not only directly affects bedtime procrastination of college students, but also influences bedtime procrastination by affecting the level of mobile phone addiction of an individual through physical activity, which verifies H2 and it is in line with the results of the previous studies [51–53]. Adolescents with a serious tendency towards mobile phone dependence will actively shift their attention to the operation and use of mobile phones and will also transfer their attention preferences, interests, and emotional experience needs to the mobile phone network while abandoning and avoiding real interpersonal interactions and physical exercise activities. Also, mobile phone addiction is often accompanied by frequent static behaviors, screen-front behaviors, sedentary behavior, etc., and mobile phone-addicted adolescents tend to spend their leisure time on excessive impulsive and dependent mobile phone use.

There is a moderate to high level of correlation between sleep quality problems and daily physical activity, and bedtime procrastination can be effectively alleviated through physical activity [54, 55]. Mobile phone addiction is a hindrance to college students’ practice of physical activity, and the more serious the addiction tendency is, the more negative the physical activity is. In essence, mobile phone addiction triggers a series of negative psychological responses (e.g., social exclusivity, depression, anxiety, etc.), leading to low-energy physical activity and inactivity [56]. The results of Chen’s study showed that the longer the duration of physical activity, the lower the incidence of excessive mobile phone use, and the lower the sleep delay associated with mobile phone addiction, suggesting the positive role of physical activity in reducing mobile phone use before bedtime [57]. Based on this, this study suggests that college students’ active participation in physical activity can alleviate the degree of individual mobile phone addiction and improve sleep procrastination.

### Anxiety mediates the relationship between mobile phone addiction and bedtime procrastination among university students

In this study, we found that anxiety plays a partial mediating role in exploring the effects of mobile phone addiction on college students’ bedtime procrastination (mediating effect size of 25.1%), i.e., mobile phone addiction increases individuals’ anxiety and thus exacerbates their sleep procrastination behaviors, which verifies H3. Firstly, we found that there is a significant positive correlation between mobile phone addiction and anxiety, i.e., individuals with a high degree of mobile phone addiction are more prone to higher levels of anxiety, which is more consistent with the results of previous studies [58–60].A higher level of mobile phone addiction affects the individual’s study and life and leads to higher anxiety [61, 62]. Previous research has shown that mobile phone addiction not only increases negative emotions such as distress, heightened anxiety, and depression but may also diminish positive emotions, affecting adolescents’ level of emotional balance. This ability to balance emotions can significantly and positively predict sleep quality and exacerbate bedtime procrastination, which is consistent with the findings of Kaiqing Tang [63] and Shan Hongbo [64] et al. There are also studies showing that sleep procrastination produces many negative outcomes, such as sleep deprivation and difficulty falling asleep, and these phenomena are also closely related to anxiety [65] Individuals with high levels of anxiety show stronger addictive behaviors, and the use of mobile phones can alleviate negative emotions, which in turn may enhance addictive tendencies [39],Therefore, schools need to focus on the mental health of college students and monitor their anxiety levels to avoid further exacerbations of negative emotions, and provide timely help and counseling to alleviate the occurrence of sleep procrastination and reduce the problem of mobile phone addiction among college students.

### Physical activity and anxiety have a chain mediating role between mobile phone addiction and bedtime procrastination in college students

This study also found that physical activity and anxiety play a mediating role between mobile phone addiction and bedtime procrastination among college students, validating H4. The self-regulation deficit model suggests [66] that people with psychosocial problems tend to lack self-control and are more likely to become addicted to uncontrolled mobile phone use, which leads to a series of problematic behaviors in reality. Compensatory Internet Use Theory suggests that when an individual encounters a psychological problem, in order to alleviate the pressure, escape from the pain, etc., they become addicted to their mobile phone. The decline in sleep quality caused by mobile phone addiction will make people tired and depressed, and serious sleep procrastination will not only trigger internalization problems (e.g., anxiety) but also interfere with normal daytime physical exercise activities [67]. The correlation results show that physical activity is significantly negatively correlated with anxiety, indicating that the higher the level of physical activity, the lower the level of anxiety, which is also in line with the results of the study by Hongbo Gu [68]. Research has shown that anxiety can indirectly affect sleep procrastination through mobile phone addiction and that physical activity, as a positive intervention, has a role to play in alleviating anxiety and reducing the level of mobile phone addiction [69]. It is important to note that there is a masking effect between the three mediating pathways, from mobile phone addiction to bedtime procrastination, in the whole model. This result indicates the complexity of the effect of mobile phone addiction on bedtime procrastination and also suggests that mobile phone addiction may not be beneficial to bedtime procrastination under any circumstances. Information behavior itself is complex, and the occurrence of smartphone behavior is affected by a variety of factors and contexts. College students’ own cultural constructs, habits, and cognitive styles, as well as the technological and social environments in which they live, all have an impact on their mobile phone use behavior.

College is an important stage for the construction of values and the development of behavioral habits, and guiding the rational use of mobile phone electronic products and curbing the proliferation of sleep procrastination are of great significance to the improvement of college students’ emotional state and the enhancement of their physical activity level. This suggests that colleges and universities can encourage college students to actively participate in physical exercise to alleviate and prevent the tendency towards mobile phone addiction. Physical exercise not only strengthens the body but also relieves anxiety, thus improving sleep quality and sleep procrastination.

## Conclusion

1. There is a significant correlation between mobile phone addiction, bedtime procrastination, physical activity, and anxiety.
2. Physical activity and anxiety partially mediate the relationship between mobile phone addiction and bedtime procrastination, respectively.
3. Physical exercise and anxiety played a chain mediating role between mobile phone addiction and bedtime procrastination, and the masking effect was also significant.

## Data Availability

The Data generated during the current study are available from the corresponding author on request.
